# Urban-rural differences in food consumption and environment and anthropometric parameters of older adults: results from ELSI-Brazil

**DOI:** 10.1590/0102-311XEN179222

**Published:** 2023-07-17

**Authors:** Nair Tavares Milhem Ygnatios, Bruno de Souza Moreira, Maria Fernanda Lima-Costa, Juliana Lustosa Torres

**Affiliations:** 1 Núcleo de Estudos em Saúde Pública e Envelhecimento, Fundação Oswaldo Cruz/Universidade Federal de Minas Gerais, Belo Horizonte, Brasil.; 2 Centro Universitário Santa Rita, Conselheiro Lafaiete, Brasil.; 3 Instituto de Pesquisas René Rachou, Fundação Oswaldo Cruz, Belo Horizonte, Brasil.; 4 Departamento de Medicina Preventiva e Social, Universidade Federal de Minas Gerais, Belo Horizonte, Brasil.

**Keywords:** Healthy Diet, Rural Areas, Eating, Fruit, Body Mass Index, Alimentação Saudável, Áreas Rurais, Consumo Alimentar, Frutas, Índice de Massa Corporal, Dieta Saludable, Medio Rural, Ingestión de Alimentos, Frutas, Índice de Masa Corporal

## Abstract

This study aimed to identify dietary and anthropometric differences in older Brazilian adults (≥ 50 years old) living in urban-rural areas. This is a cross-sectional study with data from the second wave (9,949 participants) of the *Brazilian Longitudinal Study of Aging* (ELSI-Brazil) from 2019-2021. Weekly dietary intake of fruit/vegetables, beans, and fish; self-perception of salt consumption; food environment (availability of fruit/vegetables in the neighborhood and self-production of food); and objective anthropometric parameters (body mass index [BMI] and waist circumference [WC]) were evaluated. Analyses were adjusted for schooling level. Compared to urban areas, rural areas show lower consumption of fruit/vegetables five days or more per week (74.6% vs. 86.4%) and greater adequate salt intake (96.8% vs. 92.1%) - differences we observed for men and women. Rural areas showed lower high WC (61.9% vs. 68%), significant only for men. Considering food environment, rural areas had lower fruit and vegetable availability in the neighborhood (41.2% vs. 88.3%) and higher self-production of food (38.2% vs. 13.2%). We observed a lower consumption of fruit/vegetables five days or more per week in rural areas with fruit/vegetable availability in the neighborhood and no self-production of food. Urban and rural areas show food and nutritional diversity. Incentives for fruit or vegetable consumption among residents in urban areas should consider the greater availability of these foods in their neighborhood, whereas, in rural areas, self-production of food should be encouraged. Adequate salt intake and ideal WC maintenance should be reinforced in urban areas.

## Abstract (pt)

Objetivou-se identificar diferenças alimentares e antropométricas entre adultos mais velhos brasileiros (≥ 50 anos) residentes em áreas urbano-rurais. Trata-se de um estudo transversal com dados da segunda onda (9.949 participantes) do *Estudo Longitudinal da Saúde dos Idosos Brasileiros* (ELSI-Brasil), de 2019-2021. Foram avaliados: consumo alimentar semanal de fruta/hortaliça, feijão e peixe; autopercepção do consumo de sal; ambiente alimentar (disponibilidade de fruta/hortaliça na vizinhança e produção própria de alimentos); e parâmetros antropométricos objetivos (índice de massa corporal [IMC] e circunferência da cintura [CC]). As análises foram ajustadas por escolaridade. Em comparação com as áreas urbanas, observaram-se nas rurais: menor consumo de fruta/hortaliça em cinco dias da semana ou mais (74,6% *vs.* 86,4%) e maior consumo adequado de sal (96,8% *vs.* 92,1%) - diferenças observadas para homens e mulheres. A CC elevada foi menor nas áreas rurais (61,9% *vs.* 68%), sendo significativa somente para homens. Houve menor disponibilidade de fruta/hortaliça na vizinhança (41,2% *vs.* 88,3%) e maior produção própria de alimentos (38,2% *vs.* 13,2%) nas áreas rurais. O consumo de fruta/hortaliça em cinco dias da semana ou mais foi menor nas áreas rurais quando houve disponibilidade de fruta/hortaliça na vizinhança e ausência de produção própria de alimentos. Há diversidade alimentar e nutricional entre áreas urbano-rurais. O incentivo ao consumo de fruta/hortaliça nas áreas urbanas deve considerar a disponibilidade de fruta/hortaliça na vizinhança, enquanto nas áreas rurais deve ser em conjunto com a produção do próprio alimento. O consumo adequado de sal e a manutenção da CC nos valores ideais devem ser reforçados nas áreas urbanas.

## Abstract (es)

El objetivo fue identificar diferencias alimentarias y antropométricas entre adultos mayores brasileños (≥ 50 años) que viven en áreas urbano-rurales. Se trata de un estudio transversal con datos de la segunda ola (9.949 participantes) del *Estudio Longitudinal Brasileño sobre el Envejecimiento* (2019-2021). Se evaluaron el consumo semanal de alimentos, como frutas/verduras, frijoles y pescado; autopercepción del consumo de sal; entorno alimentario (disponibilidad de frutas/verduras en el barrio y la producción propia de alimentos); y parámetros antropométricos objetivos (índice de masa corporal [IMC] y circunferencia de la cintura [CC]). Los análisis se ajustaron por escolaridad. Se observó un menor consumo de frutas/verduras en las zonas rurales respecto a las urbanas en ≥ 5 días/semana (74,6% *vs.* 86,4%), mayor consumo adecuado de sal (96,8% *vs.* 92,1%), y estas diferencias se observaron para hombres y mujeres. La CC elevada fue menor en las zonas rurales (61,9% *vs.* 68%), y fue significativa solo para los hombres. Hubo una menor disponibilidad de frutas/verduras en el barrio (41,2% *vs.* 88,3%) y mayor producción propia de alimentos (38,2% *vs.* 13,2%) en las zonas rurales. El consumo de frutas/verduras en ≥ 5 días/semana fue menor en las zonas rurales cuando hubo frutas/verduras disponibles en el barrio y ausencia de producción del propio alimento. Existe una diversidad alimentaria y nutricional entre las zonas urbanas y rurales. Fomentar el consumo de frutas/verduras en las zonas urbanas debe tener en cuenta la disponibilidad de frutas/verduras en el barrio, mientras que en las zonas rurales debe tener en cuenta también la producción del propio alimento. Se debe reforzar el consumo adecuado de sal y el mantenimiento de la CC en valores ideales en las zonas urbanas.

## Introduction

As other upper-middle-income countries, Brazil has experienced rapid urbanization since 1950’s ^1^. The 2010 *Demographic Census* showed that 84% of its population lived in urban areas ^2^. Urban population growth have reinforced social and health inequalities in urban areas ^3^. However, rural life is associated with greater poverty and worse health conditions ^4^, often related to absent economic opportunities, worsened provision of health services, and difficult access to these services ^5^.

Urbanization has also negatively affected the population’s nutritional profile. Higher consumption of ultra-processed foods and body weight gain have been observed ^6^, which have become important public health problems. Changes in food consumption may stem from the distance between individuals and primary food production, altering their eating habits. Still, these changes may be due to a lack of time to acquire and prepare meals and the greater amount of processed and ultra-processed food products in individuals’ diet ^7^.

The second edition of the *Dietary Guidelines for the Brazilian Population* of the Ministry of Health ^8^, published in 2014, recommends the preferential consumption of fresh or minimally processed plant-based foods. Thus, some food groups, such as fruits, vegetables, beans, and fish, configure healthy eating markers, which population surveys have investigated ^9^. To prevent chronic noncommunicable diseases, the World Health Organization (WHO) recommends 400g/day of fruits/vegetables and fish at least once per week ^7^. Consuming beans also contributes to the intake of protein, fiber, vitamin B, iron, zinc, and calcium and, combined with rice, constitutes an excellent protein source that is typical of the Brazilian eating habit ^8^.

However, the recommended consumption of healthy foods remains insufficient throughout Brazil if we consider adults aged 18 years or older ^9^. Previous studies with this population have indicated that the consumption of these foods is even lower in rural areas, especially of fruits/vegetables and fish, when compared to urban areas ^9^ ^,^ ^10^. International evidence found a lower dietary diversity among older adults living in the rural areas of China ^11^ and lower fruit consumption among American older adults in rural areas ^12^. On the other hand, the WHO recommends that adults consume up to 5g of salt per day at most (or 2,000mg of sodium), which tends to be higher in individuals who have unhealthy eating habits ^7^ and in urban areas, according to data from the 2019 *Brazilian National Health Survey* (PNS) ^13^.

Associated with urbanization and worsening in food consumption patterns, overweight prevalence has increased in Brazil - higher in urban areas than in rural ones and in women than in men ^6^. In contrast, underweight prevalence has increased among rural areas residents. Although both women and men show a similar underweight predominance, men’s body mass index (BMI) suffers a greater impact ^14^. However, international studies indicate a greater increase in BMI over 12 years in the rural Chinese population than in its urban population and in men than in women ^11^.

Several aspects can explain such disparities between urban and rural areas, such as health behaviors (influenced by sociodemographic, ethnic, cultural, regional, and religious factors) ^8^ and food environment. Food environment encompasses the physical, socioeconomic, cultural, and political conditions providing individuals’ diet. It can be evaluated at the community or individual level, including eating behavior, local commerce availability, difficulty of transportation, and physical access to food ^15^ ^,^ ^16^. Assessing these differences is extremely relevant to ensure food and nutrition security and the human right to adequate and healthy food (respecting traditional food cultures and practices) and to guide public policies addressing nutritional inequities. Despite the evidence of worse food consumption in rural areas among Brazilian adults, urban-rural disparities are yet to be specifically assessed in older adults.

Thus, this study aimed to find differences related to food consumption and environment and anthropometric parameters among older adults living in the urban and rural areas of Brazil.

## Methods

### Study design and population

This is a cross-sectional analysis of data from the second wave of the *Brazilian Longitudinal Study of Aging* (ELSI-Brazil), a population-based study that began in 2015 and was designed to represent the noninstitutionalized Brazilian population aged 50 years or above in Brazil’s five macrorregions.

ELSI-Brazil used a complex sampling design, to ensure the representation of urban and rural areas in the small, medium, and large municipalities of Brazil, combining stratified primary sampling units (municipalities), census tracts, and households. Urban and rural households were defined according to the Brazilian Institute of Geography and Statistics (IBGE) during its 2010 *Demographic Census*. IBGE defines the urban-rural classification of each census tract based on the municipal laws in force at the time of the census. Within the boundaries of each municipality, these laws determine an imaginary line called the “urban perimeter”. Census tracts in “urban perimeters” were termed urban areas, whereas rural areas encompassed the entire area outside this perimeter ^2^. Households were given the same urban-rural classification as the census tract to which they belong. All residents aged 50 and above in the selected households were eligible to participate in this survey ^17^, including 855 participants who needed a proxy. The second wave of the survey was conducted from August 2019 to March 2021 with 9,949 participants, of which 24% were recruited by sample replacement to continue ensuring national representativeness. More information can be elsewhere ^17^ ^,^ ^18^.

ELSI-Brazil was approved by the Research Ethics Committee at the Oswaldo Cruz Foundation (protocol n. 34649814.3.0000.5091). Participants signed an informed consent forms for all research procedures, including home interviews and physical measurements. Volunteers were guaranteed the right to refuse both answering any question in our questionnaire and undergoing any physical measurement.

### Healthy eating markers and self-perception of salt consumption

Weekly fruit or vegetable consumption (days per week) was evaluated in face-to-face interviews by the following questions: “How many days per week do you usually eat fruit?” and “How many days per week do you usually eat vegetables (such as kale, carrots, chayote, eggplant, zucchini, lettuce, and tomatoes)? Does not include potato, cassava or yams” (0-2; 3-4; or 5 days or more). Beans and fish consumption was investigated by the following questions: “How many days per week do you usually eat beans?” (0-2; 3-4; or 5 days or more) and “How many days per week do you usually eat fish?” (none; 1; or 2 days or more). This study considered all healthy eating markers available in ELSI-Brazil.

Self-perception of salt consumption was evaluated by the question: “Considering freshly prepared food and processed foods do you think your salt intake is...”. Answer possibilities included “very high”, “high”, “adequate”, “low”, and “very low”, categorized into high (very high/high) and adequate (adequate/low/very low).

### Food environment

Food environment included fruit and vegetable availability in the neighborhood, assessed by the question: “Does your neighborhood have markets, farmers markets, or other points of sale with a variety of fresh fruits and vegetables?” (not available; available) and self-production of food, investigated by the following question: “Do you and the other people who live in this household consume meat, vegetables, or fruits you grow, produce, collect, or harvest?” (does not produce; produces).

### Anthropometric parameters

Anthropometric measurements, objectively measured by standardized protocols ^19^, of body weight, height, and waist circumference (WC) were included. A Seca 813 portable digital scale (https://www.seca.com) was used to measure weight, a Nutri-Vida portable vertical stadiometer, to assess height and a Seca 201 tape measure, to gauge WC. All measurements were performed twice and their averages were used. Physical measurements of bedridden and wheelchair-bound participants were excluded.

BMI and WC were adopted as anthropometric parameters and classified according to participants’ age. BMI was obtained by dividing participants’ body weight (in kg) by their height (in m^2^) and classified according to the cutoff points recommended by the WHO for adults aged 50-59 years ^20^ (low weight: BMI < 18.5kg/m^2^, eutrophy: 18.5-24.9kg/m^2^, and overweight: > 24.9kg/m^2^) and the Lipschitz criteria ^21^ - adopted by the Brazilian Ministry of Health ^22^ - for older adults - aged 60 years or above (underweight: BMI < 22kg/m^2^, eutrophy: 22-27kg/m^2^, and overweight: > 27kg/m^2^). Regarding WC, values ≥ 80cm for women and ≥ 94cm for men were classified as high ^20^. Specific cutoff points were adopted for older adults: ≥ 88.7cm for women and ≥ 96cm for men were classified as high ^23^.

### Sociodemographic characteristics

Sex (female and male), age group (50-59; 60-69; 70-79; and ≥ 80 years), self-reported skin color (white and other, including black, mixed-race, yellow, and indigenous), marital status (married and single/widowed/divorced), and schooling level (in complete years of study, < 8; 9-11; and ≥ 12 years) were included as sociodemographic characteristics.

### Statistical analyses

First, the frequency distribution of sociodemographic characteristics according to residence in urban and rural areas was estimated and differences were verified by the Pearson’s chi-squared test with Rao-Scott correction.

Then, schooling-adjusted prevalence ratios were calculated since only this variable significantly differed between urban and rural areas (p-value < 0.001). Adjustments were performed using direct standardization. Differences between categories were evaluated by prevalence ratios (PR) and their respective 95% confidence intervals (95%CI), obtained by a schooling-adjusted Poisson regression with robust variance using residence in urban areas as a reference category.

Healthy eating, food environment, and anthropometric parameter prevalence ratios that statistically and significantly differed between households in urban and rural areas were stratified by sex, adjusted for schooling level, and plotted in graphs. As fruit or vegetable consumption can be influenced by food environments, fruit or vegetable consumption five days per week or more was also plotted according to food environment (fruit and vegetable availability in the neighborhood and self-production of food) and residence in urban and rural areas, adjusted for schooling level.

All analyses were performed using Stata/SE, version 17.0 (https://www.stata.com), employing the svy command to consider sample design complexity and individuals’ weight.

## Results

This study included the 9,949 participants of the second wave of ELSI-Brazil. Most lived in urban areas (83.8%), were females (59.3%) and had low schooling level (< 8 years, 76.1%) (Table 1). Urban areas had higher missing data than rural ones for weekly consumption of beans and fish (p < 0.05).

**Table 1:** Distribution of sociodemographic characteristics of our sample according to residence in urban and rural areas. *Brazilian Longitudinal Study of Aging* (ELSI-Brazil), 2019-2021.

Sociodemographic characteristics	Brazil (%)	Urban areas (%)	Rural areas (%)	p-value *
Sex				0.791
Female	59.3	54.3	54.9	
Male	40.7	45.7	45.1	
Age bracket (years)				0.939
50-59	30.3	47.1	47.9	
60-69	34.8	29.1	29.0	
70-79	23.1	16.2	15.8	
≥ 80	11.8	7.6	7.3	
Self-reported skin color **				0.167
White	46.5	47.6	40.2	
Other	53.5	52.4	59.8	
Marital status				0.165
Married	53.0	59.6	65.8	
Single/Widowed/Divorced	47.0	40.4	34.2	
Schooling level (years)				< 0.001
< 8	76.1	69.4	90.7	
9-11	17.1	22.4	7.2	
≥ 12	6.8	8.2	2.1	
Total (n) ***	9,949	8,339	1,610	

* p-value based on Pearson’s chi-squared test with Rao-Scott correction;

** Missing data for n = 76;

*** Number of respondents, ignoring corrections according to sampling parameters.

Brazil shows an 84.5% and 72.5% prevalence of consumption of fruit or vegetable and beans five days per week, respectively. Less than half of participants (49.2%) reported consuming fish at least once per week. Prevalence differences, adjusted for schooling level between areas of residence and in relation to healthy eating markers and self-perception of salt consumption showed that rural areas had a significantly lower prevalence of fruit or vegetable consumption five days per week or more (74.6% vs. 86.4%) and greater self-perception of adequate salt intake (96.8% vs. 92.1%) than urban areas. Considering food environments, rural areas had lower fruit and vegetable availability in the neighborhood (41.2% vs. 88.3%) and higher self-production of food (38.2% vs. 13.2%). Regarding anthropometric parameters, urban areas showed a greater prevalence of high WC than rural areas (68% vs. 61.9%) (Table 2).

**Table 2:** Prevalence of markers of healthy eating, self-perception of salt intake, food environment, and anthropometric parameters according to residence in urban and rural areas. *Brazilian Longitudinal Study of Aging* (ELSI-Brazil), 2019-2021.

Variables	Brazil (%)	Urban areas (%) *	Rural areas (%) *	PR (95%CI) **
Healthy eating markers				
Weekly consumption of fruits or vegetables (days)				
0-2	7.1	6.0	12.4	1.00
3-4	8.4	7.6	13.0	0.81 (0.59-1.11)
5 or more	84.5	86.4	74.6	0.57 (0.42-0.77) ***
Weekly consumption of beans (days) ^#^				
0-2	14.2	13.2	19.4	1.00
3-4	13.3	12.6	17.3	0.93 (0.76-1.14)
5 or more	72.5	74.2	63.3	0.75 (0.49-1.13)
Weekly consumption of fish (days) ^##^				
None	50.8	52.2	41.9	1.00
1	20.6	21.2	18.3	0.98 (0.76-1.28)
2 or more	28.6	26.6	39.8	1.36 (0.88-2.09)
Self-perception of salt consumption ^###^				
High	7.1	7.9	3.2	1.00
Adequate	92.9	92.1	96.8	2.28 (1.44-3.61) ***
Food environment				
Availability of fruits and vegetables in the neighborhood ^§^				
Not available	19.4	11.7	58.8	1.00
Available	80.6	88.3	41.2	0.17 (0.12-0.26) ***
Food self-production ^§§^				
Do not produce their own food	82.5	86.8	61.8	1.00
Produce their own food	17.5	13.2	38.2	3.31 (2.23-4.91) ***
Anthropometric parameters				
BMI ^§§§^				
Eutrophy	33.3	32.7	33.8	1.00
Underweight	9.4	8.9	11.5	1.22 (0.89-1.66)
Overweight	57.3	58.4	54.7	0.86 (0.69-1.06)
WC ^†^				
Adequate	33.4	32.0	38.1	1.00
High	66.6	68.0	61.9	0.77 (0.64-0.93) ***

95%CI: 95% confidence interval; BMI: body mass index; PR: prevalence rate; WC: waist circumference.

* Schooling-adjusted prevalence based on direct standardization;

** PR estimated by the Poisson regression model with robust variance, adjusted for schooling level; reference category: urban areas;

*** p-value p ≤ 0,05;

^#^ Missing data for n = 88;

^##^ Missing data for n = 108;

^###^ Missing data for n = 186;

^§^ Missing data for n = 69;

^§§^ Missing data for n = 53;

^§§§^ Missing data for n = 1,698;

^†^ Missing data for n = 2,071.

**Figure 1:**
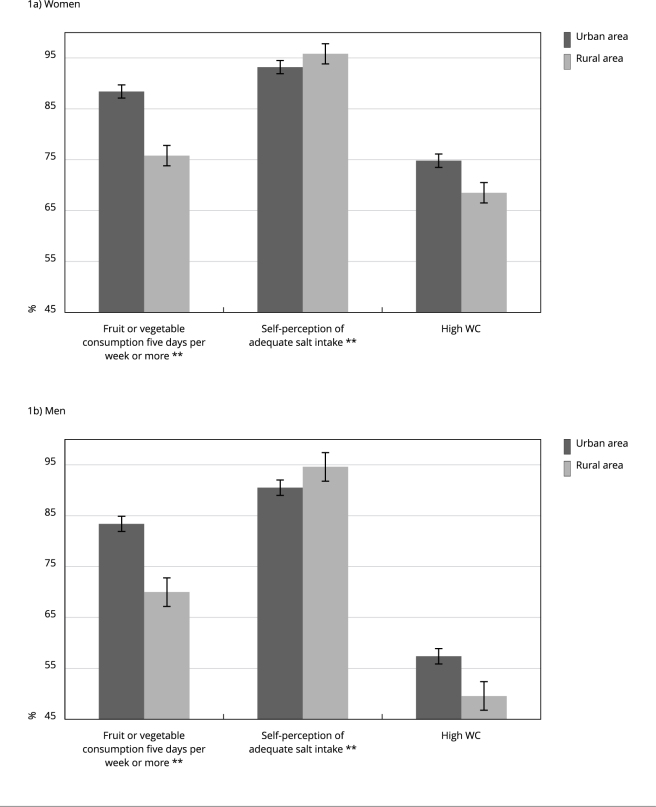
Prevalence * of fruit or vegetable consumption five days per week or more, self-perception of adequate salt intake, and high waist circumference (WC) according to residence in urban and rural areas, in women and men. *Brazilian Longitudinal Study of Aging* (ELSI-Brazil), 2019-2021.

By stratifying healthy eating markers and anthropometric parameters statistically associated with residence in urban and rural areas by sex, Figure 1 shows that rural areas had a statistically lower prevalence of fruit or vegetable consumption five days per week or more than urban areas both among women (75.8% vs. 88.4%, respectively) and men (70% vs. 83.4%, respectively). Regarding self-perception of adequate salt intake, residence areas significantly differed between males and females: higher in rural areas than in urban ones (95.8% vs. 93.2% among women and 94.6% vs. 90.5% among men). On the other hand, rural areas showed a statistically lower prevalence of high WC than urban areas only among men (49.6% vs. 57.4%, respectively).

* Prevalence adjusted by schooling level based on direct standardization; ** p-value ≤ 0.05, estimated by the Poisson regression model with robust variance, adjusted for schooling level.

**Figure 2:**
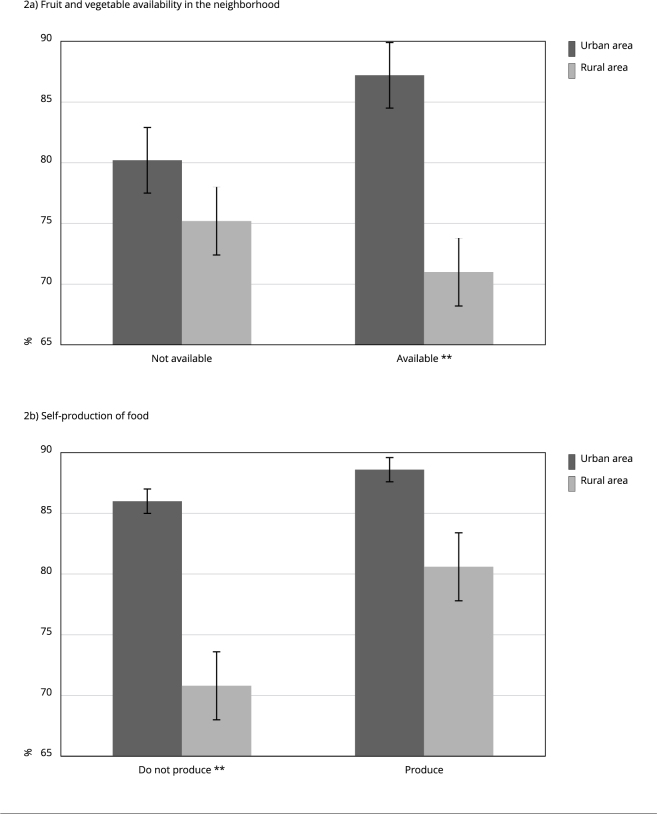
Prevalence * of fruit or vegetable consumption five days per week or more in relation to the fruit and vegetable availability in the neighborhood and self-production of food, according to residence in urban and rural areas. *Brazilian Longitudinal Study of Aging* (ELSI-Brazil), 2019-2021.

Figure 2 shows the prevalence of fruit or vegetable consumption five days per week or more in relation to residence in urban and rural areas, vegetable availability in the neighborhood, and self-production of food. Rural areas showed a statistically lower fruit or vegetable consumption five days per week (71%) than urban areas (87.2%) among participants who reported fruit and vegetable availability in their neighborhood. Those who do not produce their own food showed a statistically lower prevalence of fruit or vegetable consumption five days per week or more in rural areas (70.8%) than in urban ones (86%).

* Prevalence adjusted by schooling level based on direct standardization; ** p-value ≤ 0.05, estimated by the Poisson regression model with robust variance, adjusted for schooling level.

## Discussion

Results showed the dietary and anthropometric differences in older adults living in urban and rural areas of Brazil. Considering the healthy eating markers and self-perception of salt intake, both women and men residing in rural areas consumed less fruits or vegetables five days per week or more and had a higher self-perception of adequate salt intake. Considering food environment, rural areas had lower fruit and vegetable availability in the neighborhood and greater self-production of food. Among those who reported fruit and vegetable availability in their neighborhood, consumption of fruit or vegetables five days a week or more was higher in urban areas compared to rural areas. In case of no self-production of food, fruit or vegetable consumption five days per week or more was lower in rural areas. Finally, considering anthropometric parameters, the prevalence of high waist circumference was lower in rural areas, statistically and significantly differing only among men.

Brazil has a notably large territory, generating a climate and culture diversity reflected on the heterogeneity of its population’s eating habits. However, it still shows national culinary traditions, such as the consumption of beans ^10^. This study found a great prevalence of consumption of this healthy eating marker five days per week or more (72.5%) - a frequency that characterizes a regular Brazilian diet ^24^ -, similar to data from the 2013 PNS in older adults (aged 60 years or above) ^25^ and with no statistically differences between urban and rural areas. Also, we observed no differences between areas regarding regular weekly consumption of fish ^7^, reported by less than half of our participants (49.2%). These results differ from findings for the Brazilian adult population (aged 18 years and above) living in rural areas, who report consuming more beans and less fish ^10^. Despite the high cost of fish, its consumption requires stimulation due to its rich nutritional value and significance for the traditional Brazilian food culture ^8^.

Unlike another Brazilian study with adults and older adults ^25^, this study showed high fruit or vegetable consumption. However, rural areas showed a lower prevalence than urban areas for both sexes. This result is similar to that for the general adult population ^10^ and shows that lower fruit and vegetable availability in the neighborhood in rural areas plays a primary role in urban-rural differences since the food environment is considered strategic to ensure and to favor fruit or vegetable consumption ^26^. Previous studies have evaluated this association ^27^ ^,^ ^28^. Moreover, rural areas have a more limited access to markets specialized in the sale of fruits or vegetables and to supermarkets ^29^. However, we only observed a lower fruit or vegetable consumption in rural areas with fruit and vegetable availability in the neighborhood, showing the possible association of other factors, such as financial ones, which have been listed as determinants to increase the participation of these foods in the Brazilian diet ^30^. A study conducted in 18 countries with different income levels, including Brazil, demonstrated that rural areas require greater resource expenditure than urban areas to ensure the recommended consumption of fruits or vegetables ^31^. Notably, the consumption of these foods is important to promote health because they not only contribute to basic nutritional needs, but also play a role in reducing inflammation and preventing chronic noncommunicable diseases ^32^.

As an individual approach strategy, interventions based on food and nutrition education are needed to motivate and to improve the adoption of healthy eating habits. Such interventions should be carried out in line with the recommendations of the *Dietary Guidelines for the Brazilian Population* ^8^, which advocate respect for the biosociocultural and food diversity of the country, the valorization of the regionally available variety of fruits and vegetables, and the development of culinary skills to promote healthy eating ^33^.

This study found that those in rural areas who did not produce their own food showed lower fruit or vegetable consumption five days per week. Self-produced food, even if from a small domestic garden or community garden, can become more accessible in rural areas, compensating for the lower availability of markets. The *Dietary Guidelines for the Brazilian Population* ^8^ privilege socially and environmentally sustainable local food production systems, recognizing traditional production techniques, soil management, and minimum food processing and thus contributing to protect natural resources and biodiversity and produce safe and healthy food. In this perspective, the encouragement of family farming and rural cooperatives can favor the consumption of fruits or vegetables and, thus, benefit the health of the rural population and guarantee food and nutrition security ^10^.

In most parts of the world, production and distribution of food and dietary habits and behaviors have undergone unfavorable changes. With less time to acquire, make, and consume food (closely related to the urban way of life) comes a greater presence of ready-to-eat, processed, and ultra-processed foods in individuals’ diet who eat meals away from home and replace their main meals with snacks. These changes are reflected on the population’s nutritional profile, morbidity, and mortality ^34^. As expected for a country in nutritional transition and consistent with previous survey ^35^, we found a higher prevalence of high WC in urban dwellers, although it did not significantly differ regarding BMI. We can explain this finding by the different lifestyles of urban areas. Individuals living in urban areas generally show sedentary behavior, spending more time watching television and less time in work-related physical activities ^36^. When stratified by sex, this urban-rural disparity is even greater in males than in females ^36^, which may explain the difference in high WC found only among males in this study.

We also observed a higher prevalence of self-perception of adequate salt intake among rural area residents, as did the 2013 PNS ^37^. The interpretation of these results should consider a possible limitation of this measure since the conformity between self-perception of salt consumption and actual salt consumption remains unexplored ^38^. We should also highlight that, with aging, physiological changes can affect taste, affecting the ability of older adults to perceive salt intake ^39^. Moreover, although table salt corresponds to the main dietary source of sodium, almost 1/5 of this mineral comes from processed and ultra-processed food - present in other forms, such as monosodium glutamate, whose identification is difficult and requires knowledge -, contributing to the underestimation of salt consumption, especially in urban areas ^37^ ^,^ ^39^. Thus, the implementation of several national strategies to reduce sodium consumption is extremely important for public health (especially food and nutrition education) and to raise public awareness about the risks to health of excessive sodium consumption and guide the interpretation of nutritional labeling, especially in urban areas.

We should highlight the strengths and limitations of this study. Its strengths include the use of nationally representative and recent data on Brazilian older adults, contributing in a unique way to draw for the first time a panorama of dietary and anthropometric differences between urban and rural areas in Brazil. We also included variables that characterize participants’ food environment, which nationally based epidemiological research in aging had ignored. Such knowledge is necessary to guide national and local interventions and more effective public policies to promote healthy eating and nutrition, considering the context between the areas of residence of a society in rapid nutritional transition.

Limitations include the fact that results may be subject to memory biases due to our use of self-reported measures for most variables. The obtained information on healthy eating markers, salt consumption, and food environment suffer the influence of participants’ memories. However, the literature shows that this method of obtaining data is accurate and reliable compared to three 24-hour dietary recalls ^40^. Still, considering that people with missing data tend to show worse indicators, the higher absent data in urban areas for weekly fish consumption may partially explain the absence of differences between the areas of residence for this marker. A situation that differs from weekly beans consumption, which was slightly higher in urban areas. Moreover, individuals who consume fruits or vegetables may be more likely to know the resources available in their neighborhoods to acquire these foods and answer this question more accurately. Finally, individuals’ self-perception of their neighborhood may differ between urban and rural areas since urban neighborhoods are much better delimited.

## Conclusion

Results show a food and anthropometric diversity between urban and rural areas and that the area of residence can determine individuals’ nutritional profile. Public food and nutrition policies should target the Brazilian population as a whole but consider the singularities between urban and rural areas. Incentives for the consumption of fruits or vegetables among residents in urban areas should consider the greater availability of these foods in their neighborhood, whereas, in rural areas, it should take place with incentives for self-production of food. On the other hand, strategies to reduce salt consumption and keep waist circumference within ideal values should be reinforced in urban areas.
